# Patients’ experiences of and roles in interprofessional collaborative practice in primary care: a constructivist grounded theory study

**DOI:** 10.1017/S1463423624000148

**Published:** 2024-05-09

**Authors:** Alexandra R. Davidson, Mark Morgan, Lauren Ball, Dianne P. Reidlinger

**Affiliations:** 1 Faculty of Health Sciences and Medicine, Bond University, Gold Coast, Queensland, Australia; 2 Institute for Evidence-Based Healthcare, Bond University, Gold Coast, Queensland, Australia; 3 Centre for Community Health and Wellbeing, The University of Queensland, Brisbane, Queensland, Australia

**Keywords:** chronic disease, interdisciplinary communication, interprofessional relations, patient care team, primary health care, qualitative research

## Abstract

**Aim:**

This constructivist grounded theory study aimed to (1) explore patients’ experiences of and roles in interprofessional collaborative practice for chronic conditions in primary care and (2) consider the relevance and alignment of an existing theoretical framework on patients’ roles and based on the experiences of patient advocates.

**Background:**

High-quality management of chronic conditions requires an interprofessional collaborative practice model of care considering an individual’s mental, physical, and social health situation. Patients’ experiences of this model in the primary care setting are relatively unknown.

**Methods:**

A constructivist grounded theory approach was taken. Interview data were collected from primary care patients with chronic conditions across Australia in August 2020 – February 2022. Interviews were recorded, transcribed verbatim, and thematically analysed by (1) initial line-by-line coding, (2) focused coding, (3) memo writing, (4) categorisation, and (5) theme and sub-theme development. Themes and sub-themes were mapped against an existing theoretical framework to expand and confirm the results from a previous study with a similar research aim.

**Findings:**

Twenty adults with chronic conditions spanning physical disability, diabetes, heart disease, cancer, autoimmune, and mental health conditions participated. Two themes were developed: (1) *Adapting to Change* with two sub-themes describing how patients adapt to interprofessional team care and (2) *Shifting across the spectrum of roles*, with five sub-themes outlining the roles patients enact while receiving care. The findings suggest that patients’ roles are highly variable and fluid in interprofessional collaborative practice, and further work is recommended to develop a resource to support greater patient engagement in interprofessional collaborative practice.

## Introduction

Primary care is increasingly focused on preventing, treating, and managing chronic conditions. Almost half of Australians (47.3%) self-report living with at least one of ten chronic conditions outlined in the National Health Survey in 2017–18 (Australian Bureau of Statistics, 2019). Premature mortality and morbidity are implications of chronic conditions (Australian Institute of Health and Welfare, 2018). With nearly one in four Australians having two or more chronic conditions in 2014–15 (Australian Bureau of Statistics, 2019), the focus of primary care has shifted to address the complexity of chronic conditions and the impacts it has on individuals and communities (Australian Institute of Health and Welfare, 2018).

Chronic and multimorbid conditions ideally require a multifaceted approach to address individual’s physiological, psychological, and social care needs (Rimmelzwaan *et al*., 2020). Such an approach aligns with the interprofessional collaborative practice (IPCP) model of healthcare involving diverse health professionals (Harris and Zwar, 2014). The World Health Organization (WHO) has defined IPCP as *‘…multiple health workers from different professional backgrounds work together with patients, families, carers and communities to deliver the highest quality of care. It allows health workers to engage any individual whose skills can help achieve local health goals’.* (World Health Organization, 2010).

In Australia, primary healthcare services are predominantly through general practice, community health centres, and private allied health clinics (Australian Institute of Health and Welfare, 2018) and are funded primarily through the Australian federal government (Medicare), as well as state governments, and private health insurers (Australian Institute of Health and Welfare, 2023). One Australian government initiative to encourage IPCP in primary care is the chronic disease General Practitioner Management Plans (GPMP), which aim to facilitate comprehensive care for people with complex needs (Australian Government Services Australia, 2021). A GPMP is developed with the patient and their general practitioner (GP) and allows for two or more health professionals to be involved in the Team Care Arrangement (TCA), where an individual with a chronic condition can receive subsidised care for allied health services (Australian Government Services Australia, 2021). However, the extent to which these plans enhance IPCP in practice is unclear based on the perspectives of patients (Foster and Mitchell, 2015, Banfield *et al*., 2019) and health professionals (Cant, 2010, Jones *et al*., 2014, Orrock *et al*., 2015).

Despite the importance of including patients in IPCP, it is unknown what role patients play in IPCP in primary care. Previous studies have identified that patients want to be more readily involved in IPCP teams, including team meetings and care plan discussions (van Dongen *et al*., 2017b). Including patients’ perspectives of their care in current and future healthcare policy and research is highly recommended (Nilsen *et al*., 2006). The patient’s experience of IPCP reflects the quality of care delivered by primary health professionals, where more positive patients’ experiences reflect a higher quality of care (Donnelly *et al*., 2019). Reviews and original research looking at patient’s experiences of IPCP in primary care have recommended further investigation into the perspectives of diverse patients on the patient’s role, current and potential, in IPCP (Morgan *et al*., 2015, van Dongen *et al*., 2017b, Davis *et al*., 2018, Davidson *et al*., 2022a, Davidson *et al*., 2022b). A previous qualitative study utilised the expertise of patient advocates to develop a preliminary theoretical framework for the different roles that patients adopt throughout their care (Davidson *et al*., 2022b). The experiences and context that underpin patients’ roles are complex and require further investigation to clarify patients’ roles. Role clarification is one of the key mechanisms of successful IPCP as outlined in the WHO ‘Framework for action on interprofessional education and collaborative practice’ (World Health Organization, 2010), without understanding each team member’s role in healthcare, and IPCP cannot be adequately conducted. Therefore, the current study aimed to explore patients’ experiences of and roles in interprofessional collaborative practice for chronic conditions in primary care. A secondary aim was to consider the relevance and alignment of an existing theoretical framework on patients’ roles developed from the experiences of patient advocates with individuals with lived experience of living with a chronic condition.

## Methods

### Study design

This qualitative constructivist study adopted a lens where individuals’ views were built on their lived experiences with primary healthcare and IPCP (Crotty, 1998). Charmaz’s constructivist grounded theory (Charmaz, 2006) guided the inductive exploration of patients’ experiences and perceived roles in IPCP of chronic conditions in primary care. Themes were derived from the data provided by patients who shared their lived experiences and interactions with their world, including their primary healthcare interactions (Charmaz, 2006). Prior to the commencement of the project and at regular intervals, authors met to discuss their position within the research in a reflexivity meeting, including discussion about the authors’ dual health professional and researcher roles. The Standards for Reporting Qualitative Research were used to develop this manuscript (See Supplementary File 1).

### Participants and setting

Adults (≥18 years of age) who self-identified as living with at least one chronic condition and who primarily experience their management and treatment in Australian primary care settings were recruited through theoretical and snowball sampling (Charmaz, 2006). Theoretical sampling occurred through recruitment of participants identified as data-rich (Charmaz, 2006), from a previous study with patient advocates (Davidson *et al*., 2022b). Additionally, recruitment was conducted through the authors’ professional networks and non-government organisations that support individuals with chronic conditions, including Asthma Australia (Asthma Australia, 2022) and Consumers Health Forum of Australia (Consumers Health Forum of Australia, 2022). The chronic condition inclusion criteria were defined as a condition impacting one’s physical or mental health that has lasted or is expected to last for greater than six months (Australian Government Services Australia, 2021). Primary care was defined as any healthcare setting an individual could access at a first-contact stage, including general practice, community health centres, allied health practices, and care delivered in the home by primary healthcare professionals. The study aimed to recruit a range of people from diverse backgrounds, genders, age groups, and a range of chronic conditions were included. Once a prospective participant was identified, AD contacted the potential participant via email with the participant information sheet.

### Data collection

Due to changing directives for the global pandemic, face-to-face, telephone, and videoconference, via Zoom (Zoom Video Communications, 2021) were used to collect data. Previous research has identified telephone interviews and videoconference as appropriate qualitative data collection tools (Ward *et al*., 2015, Archibald *et al*., 2019). Use of telephone instead of Zoom was necessary where participants were unfamiliar with the technology. A semi-structured interview guide (see Supplementary File 2) was developed using key literature on IPCP, (World Health Organization, 2010, van Dongen *et al*., 2017a, van Dongen *et al*., 2017b) and the insights from the focus group study with patient advocates (Davidson *et al*., 2022b). Two members of the public, who were later participants, partnered in developing the semi-structured interview guide. The interview guide was piloted with a third member of the public who did not meet the inclusion criteria of being primarily managed in primary care settings as her specialist medical professional managed her care. The interview guide was adapted based on feedback from the three members of the public and amended during data collection, where necessary.

One-hour interviews with 20–30 participants were deemed most likely to sufficiently meet the research aims (Robinson, 2014). However, interviews were conducted until theoretical saturation was deemed to have been reached after 15 interviews, and the additional five interviews provided no new theoretical insights to support theory development (Charmaz, 2006). Interviews continued to obtain diversity in the sample, including gender, age, and condition. Interviews were audio-recorded using a tape recorder for face-to-face and telephone interviews and the in-built recording software with Zoom (Zoom Video Communications, 2021).

### Data analysis

Data analysis commenced as soon as possible following each interview (Charmaz, 2006, Robinson, 2014). Before analysis, interview recordings were transcribed verbatim by AD manually or using NVivo transcription software: https://lumivero.com/products/nvivo-transcription/ (QSR International Pty Ltd, 2020) and were then checked for accuracy by re-listening which also enabled data immersion. Charmaz’s (Charmaz, 2006) five-step analysis process was used to create inductive themes and sub-themes: (1) Initial line-by-line coding: coding that was closely related to the data, coded by AD and reviewed by DR. (2) Focused coding: direct, selective, and conceptual codes were formed based on initial codes, conducted by AD and reviewed by DR. (3) Memo-writing: notes taken during interviews and codes from steps 1 and 2 were reflected upon by AD to compare data. (4) Categorisation: Focused codes and memos collapsed into categories for synthesis, conducted by AD and DR. (5) Theme and sub-theme development: categories collapsed further to develop inductive sub-themes and themes facilitated by research team discussions. Themes and sub-themes were then mapped against the theoretical framework from a preceding study that explored the patient’s role in IPCP in primary care from the perspective of patient advocates (Davidson *et al*., 2022b), in order to test the theoretical framework from the collective view to the individual. Mapping to the previous framework was conducted to test or check and then to expand and confirm the theoretical framework of the patient’s role. Furthering data collection to follow a studied phenomenon follows Charmaz’s guidance for conducting constructivist grounded theory research (Charmaz, 2006). Microsoft Word and Excel were used to facilitate and record data analysis.

### Ethics, consent, and permission

The Bond University Health Research Ethics Committee provided ethical approval for this study (Ethics approval number: AD02910). Informed consent was obtained from participants prior to being interviewed and confirmed verbally at the start of the interview. Any identifiable information was anonymised during transcription of audio recordings.

## Results

**Figure 1.**
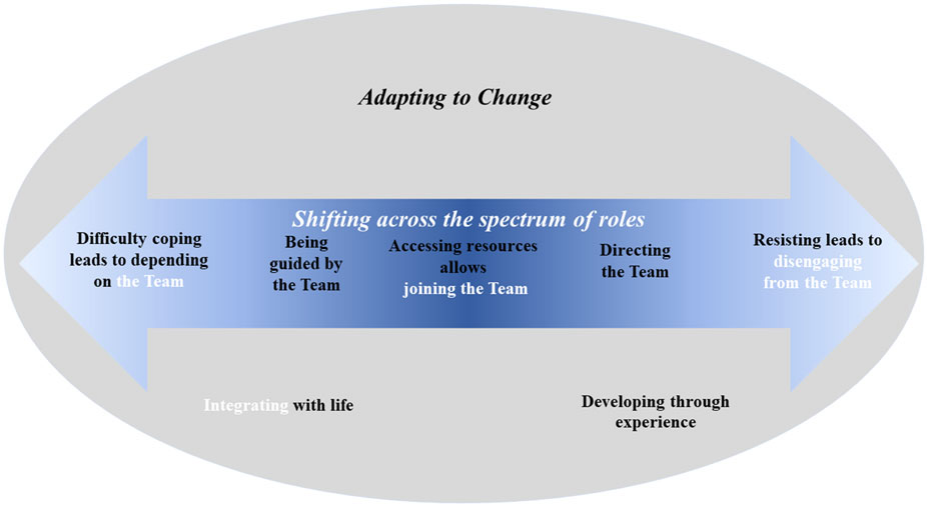
Theoretical framework of patient’s roles in interprofessional collaborative practice of chronic conditions in primary care from the perspective of patient advocates aligned with individual lived experience of people with chronic conditions. Key: Black text is the results from individual interviews with people with chronic conditions. White text is the preliminary framework from focus groups with patient advocates (Davidson *et al*., 2022b). Themes are in italics. Image is available on Figshare: (Davidson *et al*., 2022).

Twenty adults participated in an interview, to explore their experiences of IPCP and their role, current and potential, in IPCP. Interviews were conducted from August 2020 – February 2022 and ran for an average of 69 min (38 – 98 min). The demographic characteristics of participants are shown in Table 1. Participants represented a broad range of age groups, healthcare funding sources, cultural backgrounds (Australian-born, First Nations, European, Middle Eastern, and Asian), and conditions, including physical health conditions, disability, and mental illness. Participants, particularly those without healthcare training, did not naturally use the term ‘Interprofessional Collaborative Practice’ when referring to their healthcare and related professional teams. Instead, they used various terms, such as ‘team’ and ‘team care’. Figure 1 illustrates the themes and sub-themes, constructed from participant interviews, that comprise the theoretical framework of patients’ experiences and roles in IPCP in primary care for chronic conditions.

**Table 1.** Participant characteristics of patients with chronic conditions, key interprofessional collaborative practice team members, and healthcare funding stream

No.	Gender, age	Chronic condition(s)^*^	Key IPCP team members^**^	State	Private, public, or concession healthcare funding
1	W, 28	**Anxiety, BPD, Depression,** OCD	GP, Mental Health Nurse, Previous Psychiatrist, Psychologist, Receptionists,	QLD	Public
2	W, 72	**Addison’s Disease,** Hashimoto’s	Endocrinologist, GP, Massage Therapist, Receptionists	NSW	Private health cover
3	W, 79	**Asthma, T2DM,** Depression, **Hip-replacement,** HTN	Dietitian, Exercise Physiologist, GP, Nurse, Physiotherapist,	QLD	Concession – pension card
4	M, 79	**Bowel Cancer, GORD,** HTN, **Quadruple Heart Bypass**	GP, Nurse, Pharmacist, Receptionists, Student Dietitian	QLD	Concession – pension card
5	M, 60	**T1DM,** Glaucoma, **Hepatitis C**	Endocrinologist, previous GP, Podiatrist	QLD	Public
6	W, 35	Ehlers Danlos Syndrome	GP, Physiotherapist	QLD	Private health cover
7	W, 27	Coeliac Disease, **Rheumatoid Arthritis**	GP, Herself – as exercise professional, Previous Physiotherapist, Rheumatologist,	NT	Private health cover
8	W, 28	**T1DM,** Chronic postural pain	Endocrinologist, GP, Nurse, Physiotherapist	QLD	Private health cover and uses CDM
9	W, 33	Anxiety, **PCOS,** Irritable Bowel Syndrome	GP, Psychologist	QLD	Private health cover
10	W, 79	**Osteoarthritis, Osteopenia,** Sleep apnoea, T2DM	GP, Group exercise instructor, Physiotherapist, Podiatrist	NSW	Aboriginal Medical Services
11	M, 24	Eosinophilic esophagitis	Dietitian, Endocrinologist, GP, Mind-Gut Psychologist	QLD	Private health cover and parents
12	M, 20	T1DM	Diabetic Educator, Endocrinologist, GP, Nurse	QLD	Private health cover, concession card, and parents
13	M, 37	Spinal cord injury	Himself, GP, Mother – formal carer, OT	QLD	NDIS
14	M, 26	**ADHD, ASD**	GP, Psychiatrist, Psychologist	QLD	Public health cover and parents
15	M, 70	Prostate Cancer, **T2DM**	Previous Dietitian, GP, Endocrinologist, Nurse, Podiatrist, Receptionists, Urologist	WA	Concession – pension card
16	W, 20	Depression, **Lupus**	GP, Rheumatologist, Uni counsellor	QLD	Private health cover and parents
17	W, 48	**Asthma**, Bipolar	Counsellor, GP, Massage therapist, Nurse, Receptionists, Physiotherapist, Previous Psychiatrist,	NSW	Public
18	W, 60	Coeliac Disease, **Sjogren’s Syndrome**	Previous Dietitian, Endocrinologist, GP, Physiotherapist, Rheumatologist	QLD	Private health cover
19	W, 28	**ADHD**, Anxiety, **ASD**, Gender Dysphoria	GP, Psychiatrist, Psychologist	QLD	Private health cover and parents
20	M, 37	Acquired Brain Injury	GP, OT, Physiotherapist, Psychologist	VIC	Transport Accident Commission

* Condition(s) discussed in the interview with the conditions discussed the most in interviews are **bolded.**

** Listed alphabetically.

ADHD = Attention Deficit and Hyperactivity Disorder; ASD = Autism Spectrum Disorder; BPD = Borderline Personality Disorder; CDM = Chronic Disease Management plan; GORD = Gastro-Oesophageal Reflux Disease; GP = General Practitioner; HTN = Hypertension; NDIS = National Disability Insurance Scheme; OCD = Obsessive Compulsive Disorder; OT = Occupational Therapist; PCOS = Polycystic Ovarian Syndrome; T1DM = Type 1 Diabetes Mellitus; T2DM = Type 2 Diabetes Mellitus.

### Theme 1: adapting to change

Participants described that their overarching role in IPCP was to adapt to change. This adaptation stemmed from a recognition that change was part of their diagnosis and their ongoing care with an IPCP team. Participants provided examples of change they needed to accept and adapt to, including change in health professionals, services, progression through their condition, a new diagnosis, and other aspects of life that impacted their management, such as work, study, and relationships, such as becoming a parent. Incorporating health technologies in the management of their condition was also a change and an adaptation participants faced. For example, since the onset of the COVID-19 global pandemic, many participants adapted to using telehealth in their care. Another example was using insulin pumps and continuous glucose monitors for T1DM management:

*‘I was a bit lost for a bit trying to figure out what was going on and adapted to having a pump on me for a bit, and it like happened during [school] term as well’.* – (Male, 21, T1DM)

There were two predominant responses that participants described when faced with change: first, learning to accept the change and acknowledging change was part of their role and second, resisting change. Resistance came from discomfort with change or an inability due to confounding factors, such as the inability to afford to change medications or the perceived effort to see a new allied health professional as part of their IPCP care:

*Participant: ‘My sister has since found a [new health professional] in [town name] who’s apparently very good… But, you know, it’s a new [health professional] and, I’ve got enough of them, thanks’.*

*Interviewer: ‘Do you find that because you have not just the one condition, but multiple conditions having so many professionals, is that overwhelming?’*

*Participant: ‘Yes, keeping them straight. Remembering, you know, what I’m due for’.*

*Female, 60, Sjogren’s Syndrome and Coeliac Disease*

## Sub-theme 1.1: integrating with life

Participants described that their patients’ roles were conducted not only in healthcare settings but across all aspects of life, including their workplace, education institution, home, and within their communities. Participants juggled their role and integrated their patients’ role as part of their life roles. The patient’s role to engage with the IPCP team was heavily dependent on other roles they play within their life. For example, one participant prioritised her role as a mother, where she cared for her young daughter with autism, often over her management:

*‘…it [condition management] takes up too much time every day, too expensive and the mental load. Just too many things to do. Cause that’s just me, I’m also doing all of [daughter’s name] disability support at the same time’.* – (Female, 35, Ehlers Danlos Syndrome)

## Sub-theme 1.2: developing through experience

Participants described realising ‘who they were’ as patients and their roles in their care as their journey progressed over time. Participants had other foundational aspects of fulfilling these roles, such as understanding their condition, self-managing, self-empowering, and paying to feel ‘normal’, where participants described the cost of managing their condition as large. Once they felt comfortable and familiar with their interpretation of their role, they contributed to the broader health system, such as through patient advocacy.

Ongoing learning in their role occurred as participants took responsibility for themselves and were open to continue developing themselves. Taking responsibility was reflected as self-managing, empowering themselves with knowledge and control they gained by accepting that it is them who could do such things:

*‘…I think when you really take control, it gives you more…you just feel more engaged in what’s going on, and you feel like a bit more responsibility, which is a good thing, I think because then you’re doing it for you. You’re not doing it for anyone else, and that makes a lot of difference’.* – (Male, 26, ADHD and ASD)

### Theme 2: shifting across the spectrum of roles

This theme describes the five key roles constructed from the experiences of individuals with chronic conditions. Two of these roles are unique to this study, and three have been adapted from the previous qualitative study (Davidson *et al*., 2022b).

## Sub-theme 2.1: difficulty coping leads to depending on the team

Participants described a feeling of finding it difficult to cope during certain phases of their healthcare journey. In this role, participants interacted with and relinquished control to their IPCP healthcare team to get by. Individuals who described this feeling of needing to get by were pre-diagnosed, had co-morbid conditions and life impacts, or did not have a comprehensive management plan. During this time, they relied on and depended on their support system to feel like they were managing.

When participants expressed lacking the confidence or knowledge to contribute to IPCP, they reported complying with their health professionals’ instructions with minimal to no input from them as an individual. Participants felt they had a duty to respect all the decisions of their health professionals and follow guidance without question, despite the participant struggling. This unquestioning compliance was described by participants in rural and remote locations with limited access to other professionals and older persons:

*‘So, she [GP] did call for an X-Ray and possibly an ultrasound. And she came back and said, [name], there’s no cartilage. So, you just gotta [sic] live with it, alright? So, I did. Yes, doctor’.* – (Female, 80, multimorbidity)

## Sub-theme 2.2: being guided by the team

In certain situations, participants described a need to be guided by others in their care. The individual was still making choices and decisions; however, these decisions were guided by the health professionals involved in their care. An example was a young woman with psoriatic arthritis. Before diagnosis, she relied heavily on her GP to guide her through referrals and tests until they reached diagnosis together. This guidance required a sense of trust and openness to depend on others. Another example was a man with an acquired brain injury (ABI), who would set his own goals. However, how he went about achieving those goals was primarily guided by his health professionals:

*‘I had goals that I wanted to achieve, and they helped me achieve them. How we went about achieving them was up to them. Not up to me’. –* (Male, 37, ABI)

## Sub-theme 2.3: accessing resources allows joining the team

Participants were able to recognise available resources to fulfil their role adequately and utilised them when they perceived they needed to. Resources included an ability to map the different aspects of their care, having effective communication skills, and accessing the different members of their IPCP team, including their health professionals, loved ones, friends, and their community. Collaboration between these different members of their IPCP team was also important to participants. Engaging with a community of people with a similar condition where they shared management challenges with those who could relate was essential to participants. For example, a 60-year-old man who joined a cycling team commented:

*‘…you’ve got a group of people whose perspective is the same, they’ve all got diabetes, they’ve all seen an 18 [mmol/L, high blood sugar level] before, and they all know how it feels… and being in that kind of environment and when you’ve also got something like a really tough bike ride… it’s really team-building stuff’.* – (Male, 60, T1DM)

Aspects of their care included appointments, having health professionals they can trust and identifying broader health and social care resources, such as appointment transportation services and organisations that provide additional services and education. Individuals gained communication skills as they progressed through their healthcare journey, such as being open to give feedback to health professionals when experiences did not align with their expectations.

## Sub-theme 2.4: directing the team

Participants described that in some situations, they would direct their care, including navigation of services, and coordinating multiple health professional appointments. However, those with higher literacy skills and those with a strong support network, including involved parents or partners, directed their care with more confidence. At other times, directing care was necessary due to a perceived lower quality IPCP, such as when participants did not think that health professionals were communicating.

Directing care also involved a proactive approach, where participants would seek out management options that would reduce the progression of their condition and the development of co-morbidities. An example was an Aboriginal woman who followed up on her annual vaccinations and participated in group exercise classes to reduce her risk of future falls. Directing included asking questions and for second opinions when dissatisfied with answers to ensure that their care was the best for them and their situation:

*‘…well, what’s the other options? And exploring the lesser of the two evils? And maybe I don’t want to go on that drug… Is there a better option for me? And not just accepting it as well. This is the gold standard. This is what we have to do. Like well, does the gold standard work for me? Does it work in my situation?’ –* (Female, 27, psoriatic arthritis and coeliac disease)

## Sub-theme 2.5: resisting leads to disengaging from the team

Participants described resistance as a craving to be independent and not rely on a team of health professionals, often driven by a prior negative healthcare experience. Denial of the need for health professional input underpinned disengaging or resisting. When individuals attempted to connect with health professionals, they often relapsed if their expectations were unmet. However, disengaging from health professionals did not mean that the person was disengaged from their condition, as described by this man with T1DM:

*‘…I don’t see health professionals as much as other people do, I know that. But it’s not that I haven’t tried, and I’ve had experiences, you know, it’s not because I am not engaged with my health in any way. I look after all these things’.* – (Male, 60, T1DM)

## Alignment with existing theoretical framework

The themes and sub-themes developed were adapted from an earlier theoretical framework (Davidson *et al*., 2022b), which was developed from focus groups with patient advocates. The key changes which were made to the original theoretical framework as a result of the current study’s focus on patients with lived experience of chronic conditions are now briefly summarised. See Supplementary File 3 for Phase 1 vs Phase 2 figure comparison.

The original theoretical framework was broadly confirmed by patients. However, an additional theme and sub-themes were developed and added to the framework, and one theme was removed, to reflect the deeper perspectives arising from those with lived experience. Theme 1 ‘Adapting to change’ was added to the theoretical framework to reflect the participants’ experiences with moving along the spectrum described in Theme 2. The additional sub-themes developed were specifically, ‘Being guided by the Team’ and ‘Directing the Team’. The original three sub-themes were adapted to reflect the precursory actions that participants engaged in to fulfil the role, for example, ‘Difficulty coping leads to depending on the Team’. replaced ‘Relinquishing control to the Team’. Further, two sub-themes were redefined in response to participant’s lived experience: firstly, the sub-theme ‘Developing through experience’ replaced the original sub-theme ‘Learning about their role’ as this more aptly reflected how participants changed to be equipped for the role and secondly, the sub-theme ‘Integrating with life’ replaced ‘Integrating patient’s role with life roles’. As this sub-theme was interpreted to encompass the original Theme 1 ‘Juggling roles’, this theme was removed.

## Discussion

This study has achieved two aims: it has explored the experiences of patients with chronic conditions and their roles regarding IPCP in primary care and has advanced an existing theoretical framework developed from the experiences of patient advocates (Davidson *et al*., 2022b). The additional two patients’ roles constructed from this study, ‘Being guided’ and ‘Directing care’, highlight the diversity of the spectrum of patients’ roles in IPCP as viewed by the patients themselves. Additionally, there are key members of a patient’s IPCP team outside of the traditional health professions, including non-health professionals such as family, carers, and formal and informal support groups.

Although not all patients engaged with their IPCP team, they did express a clear appreciation for input from multiple professionals and their social supports, including family and friends, in their care to maximise healthcare outcomes. Those who were included were able to experience the benefits of being integrally involved in their own care, such as a sense of empowerment to self-manage, and feeling supported by a team. This aligns with other studies with similar research aims, including a qualitative focus group study exploring patients’ perspective of primary care interprofessional team meetings (van Dongen *et al*., 2017a). Participants wanted to be included in team discussions as they wished to be included in decision-making. Additionally, previous studies have described patients have a ‘patient system’ which include the patient, caregivers, and family members (van Dongen *et al*., 2017a, Grol *et al*., 2020, Quigley *et al*., 2021, Davidson *et al*., 2023). The results from the current study also highlight that participants wish for health professionals to collaborate with their non-health professional team members including their family and social supports. This can inform health professionals’ practice in primary care.

A patient’s ideal role in IPCP was found to fluctuate according to external factors such as a new diagnosis, disease progression, or sociocultural transitions as well as individual preferences. These may include the ability of health services to provide accessible care and alternative service delivery methods (Song *et al*., 2019). This means there are likely some occasions when a patient will prefer to be guided, but on other occasions they may prefer to be directing their own care. Patient-centred care supports the notion that an individual should be able to choose the level of their engagement at each healthcare interaction, regardless of their previous engagement. Health professionals’ perceptions of the role of the patient may differ between professions (Pullon *et al*., 2011), but it is incumbent on them to support patients to be involved in shared decision-making, implement education provided by professionals, and develop self-management skills (Dineen-Griffin *et al*., 2019). The notion that patients will disengage from IPCP does not align with the ‘ideal’ role described by health professionals in previous studies. However, Stewart’s definition of patient-centred care, that ‘the patient should be the judge of patient-centred care’ (Stewart, 2001), is important when considering patients’ roles in IPCP. Therefore, allowing, and even enabling, the patient to be disengaged from IPCP care could be patient-centred if the patient wishes to be disengaged due to preference. However, if patients disengage because of a previous negative healthcare interaction, health professionals should explore the reasons behind disengagement. Regardless of the reason behind patient disengagement, health professionals should explore opportunities to support disengaged patients to engage and connect with the team when the time appropriately suits the patient in the future.

Self-management and shared decision-making are a key part of chronic disease management, fitting mostly within the ‘Accessing resources’ role defined in the current study. Understanding how self-management varies depending on the individual and disease trajectory and that interactions with healthcare professionals directly impact a patient’s ability to implement self-management, is vital for all IPCP team members, including the patient (Niño de Guzmán Quispe *et al*., 2021). Additionally, shared decision-making is described as a key role for the active patient’s role in IPCP care. In an IPCP team, communication and shared decision-making have been debated as a time-intensive process (Blair and Légaré, 2015, Oandasan *et al*., 2009). There are several developed tools that may assist health professionals in implementing shared decision-making in a IPCP team environment (Stacey *et al*., 2010, Légaré *et al*., 2011). However, further development and validation of these tools are needed to identify whether they improve IPCP care (Stacey *et al*., 2010).

An ability to implement self-management strategies, remain autonomous, and contribute to shared decision-making is not always feasible (Jin *et al*., 2021). In the event of impairment, severe illness, and emergencies, such as some examples described by participants in this study, patients may not have capacity to fulfil their preferred role of an active team member (van Dongen *et al*., 2017a). Transparently encouraging patients to accept guidance and support from health professionals, when they need to relinquish control to the team, is vital to ensure that patients still receive appropriate levels of care. Health professionals can also remind patients that relinquishing decision-making to their IPCP team may only be temporary.

It is important for health professionals to be aware of the range of roles that patients could fulfil and to understand that the roles performed will shift and evolve according to changes in health and other life impacts. Identification of where an individual sits on the spectrum of roles, and the key team members engaged in an individual’s IPCP team is vital to better understand the best role for each patient and the support they may need. To facilitate this, it is recommended that a resource is used by patients and health professionals to identify patients’ roles and preferences in IPCP in primary care and reviewed regularly. However, existing tools may not fit with the current study’s framework of patients’ roles, which is inclusive of all chronic conditions and is IPCP-focused.

Current tools developed for IPCP are targeted and validated with health professionals, including the Assessment of Interprofessional Team Collaboration Scale (Orchard *et al*., 2018), Assessment for Collaborative Environments (Tilden *et al*., 2016), and Collaborative Practice Assessment Tool (Schroder *et al*., 2011). These tools assess the level of collaboration from the health professional perspective only. Although these tools may include patient engagement statements or questions (Schroder *et al*., 2011, Tilden *et al*., 2016, Orchard *et al*., 2018), the tools do not assess the patient’s preferred role and are not designed or validated to be completed by the patient themselves. Additionally, these tools were validated in a variety of healthcare settings, such as tertiary hospital settings, where there are more opportunities and enablers for IPCP (Davidson *et al*., 2022a). Although applicable to primary care, a resource that is specific to the setting may be required.

Broader tools developed for patients to self-report their level of involvement in care are also available. However, these consist of minimal items around team care, mostly focus on self-management and interactions with sole practitioners (in particular doctors), and are also not primary care specific (Kearns *et al*., 2020). These tools include the Patient Activation Measure (PAM), which has been used to measure patients’ outcomes in intervention studies (Hibbard *et al*., 2004). As a concept, patient activation has been shown to improve patients’ health outcomes (Hibbard *et al*., 2007, Hibbard *et al*., 2009). Patient activation is a behavioural concept that relates to the extent that a patient understands the role they play in their own care and their capacity to fulfil the said role (Hibbard *et al*., 2007). However, the PAM tool in its current form only relates to a patient’s confidence, skills, and knowledge to play an active role in their care and self-management (Hibbard *et al*., 2009). It does not include the person’s confidence, skills, or knowledge to play a role in IPCP and how they interact with a team of professionals.

### Strengths and limitations

Participants from this study were not required to be currently receiving structured IPCP to be interviewed. Both participants and health professionals from different disciplines will have different understandings of what constituted IPCP even when using the same definition. For example, structured programmes of IPCP may be viewed as the only kind of IPCP and are preferred by patients (Davidson *et al*., 2022a). Others see less structured programmes, such as the GPMP/TCA plans funded by Medicare are also IPCP. However, the ongoing Medicare funding issues for these plans may result in poorer IPCP. Therefore, structured IPCP is not hardwired into Australian primary care and thus was not an inclusion criterion in this study.

The diversity in the sample of individuals interviewed, including gender, age groups, and chronic conditions, was a strength of this qualitative study. However, although there were no exclusion criteria around diverse groups, one Aboriginal woman, and one self-identified transgender woman participated in this study. The theoretical framework captured their experiences which are unique to their circumstances; however, more diverse groups such as Aboriginal and Torres Strait Islander people and people in the LGBTQIA+ communities have been explored in similar settings (Klein *et al*., 2018, Wilson *et al*., 2020). A limitation of this study was the exclusion of paediatric patients. Interprofessional collaborative practice of paediatric cases is known to be more readily implemented as family-centred approaches are common practice (Platt *et al*., 2018).

Another limitation is the timeframe that interviews were held over a period of 18 months. During the 18-month timeframe, the Australian population experienced varying levels of impact of the COVID-19 pandemic. The impacts on the participants’ healthcare experiences during this time have been considered and outlined where applicable in the results.

An additional strength of this study was the interviewing style, where the interviewer, AD, built rapport with participants. A key question, added through the piloting stage of the interview guide, was ‘Checking In – How do you feel after the interview?’. Participants responded positively to this question, outlining that they appreciated the opportunity to share their experiences and thanked the interviewer for taking the time to listen.

## Conclusions

The understanding of patient’s roles was expanded by building on the theoretical framework from a previous study with patient advocates on patient’s roles in interprofessional collaborative practice (Davidson *et al*., 2022b), with the addition of the individual lived experience. Given the lack of a patient-led resource to guide the role of the patient in IPCP, it is recommended that future research focus on the development of such a resource. Ideally, the resource should support patients and health professionals to identify if this role needs to change to improve outcomes and how to support patients to move along the spectrum to their preferred role. Further, future research should identify which patients’ role or roles across the spectrum of roles produces better outcomes, for patient experience as well as the other three aims of healthcare: improved health professional experience, reduced healthcare costs, and improved health outcomes.

## Supporting information

Davidson et al. supplementary material 1 Davidson et al. supplementary material

Davidson et al. supplementary material 2 Davidson et al. supplementary material

Davidson et al. supplementary material 3 Davidson et al. supplementary material

## Supplementary material

To view supplementary material for this article, please visit https://doi.org/10.1017/S1463423624000148
