# Supplementary material for: Patients’ experiences of and roles in interprofessional collaborative practice in primary care: a constructivist grounded theory study
Source: Prim Health Care Res Dev. 2024 May 9;25:e24. doi: 10.1017/S1463423624000148 (PMC11091539; doi:10.1017/S1463423624000148)
Supplement: Davidson et al. supplementary material 1 — Davidson et al. supplementary material [file S1463423624000148sup001.docx]

Article Title: Patients’ experiences of interprofessional collaborative practice in primary care: a constructivist grounded theory study

Supplementary File 1: Standards for Reporting Qualitative Research (SRQR) Checklist

Reference: O'brien, B. C., Harris, I. B., Beckman, T. J., Reed, D. A. & Cook, D. A. 2014. Standards for reporting qualitative research: a synthesis of recommendations. Acad Med, 89, 1245-51.

| No. | Topic | Item | Manuscript Page and Line Number |
| --- | --- | --- | --- |
| **Title and Abstract** | | | |
| S1 | Title | Concise description of the nature of and topic of the study identifying the study as qualitative or indicating the approach (e.g., ethnography, grounded theory) or data collection methods (e.g., interview, focus group) is recommended | Title Page |
| S2 | Abstract | Summary of elements of the study using the abstract format of the intended publication; typically includes background, purpose, methods, results, and conclusions | Page 1, line 1 - 24 |
| **Introduction** | | | |
| S3 | Problem formulation | Description and significance of the problem/phenomenon studied; review of relevant theory and empirical work; problem statement | Page 2, line 27 – Page 4, line 76 |
| S4 | Purpose or research question | Purpose of the study and specific objectives or questions | Page 4, line 76 - 81 |
| **Methods** | | | |
| S5 | Qualitative appoach and research paradigm | Qualitative approach (e.g., ethnography, grounded theory case study, phenomenology, narrative research) and guiding theory if appropriate; identifying the research paradigm (e.g., postpositivist, constructivist/interpretivist) is also recommended; rationale | Page 4, line 84-90 under Study Design sub-heading |
| S6 | Researcher characteristics and reflexivity | Researchers’ characteristics that may influence the research, including personal attributes, qualifications/experience, relationship with participants, assumptions, and/or presupposition; potential or actual interaction between researchers’ characteristics and the research questions, approach, methods, results and/or transferability | Page 4, line 90-92 |
| S7 | Context | Setting/site and salient contextual factors; rationale | Page 5, line 105-108 |
| S8 | Sampling strategy | How and why research participants, documents, or events were selected; criteria for deciding when no further sampling was necessary (e.g., sampling saturation); rationale | Page 4, line 94-105, and Page 6, line 128-132 |
| S9 | Ethical issues pertaining to human subjects | Documentation of approval by an appropriate ethics review board and participant consent, or explanation for lack thereof; other confidentiality and data security issues | Page 7, line 157 – 162 Ethics, consent, and permission sub-heading and Page 21, line 486-488, Ethical standards |
| S10 | Data collection methods | Types of data collected; details of data collection procedures including (as appropriate) start and stop dates of data collection and analysis, iterature process, triangulation of sources/methods, and modification of procedures in response to evolving study findings; rationale | Page 5, line 112 – Page 6, line 134– Data Collection sub-heading |
| S11 | Data collection instruments and technologies | Description of instruments (e.g., interview guides, questionnaires) and devices (e.g. audio recorders) used for data collection; if/how the instrument(s) changed over the course of the study. | Page 5, line 117- Pgage 6, line 126 and Supp File 2 |
| S12 | Units of study | Number and relevant characteristics of participants, documents, or events included in the study; level of participation (could be reported in results) | In results, Page 7, line 164- 170 and Table 1 |
| S13 | Data processing | Methods for processing data prior to and during analysis, including transcription, data entry, data management and security, verifcation of data integrity, data coding, and anonymization/deifendification of excerpts | Page 6, line 1136-140 |
| S14 | Data analysis | Process by which inferences, themes, etc., were identified and developed, including the researchers invovled in data analysis; usually references a specific paradigm or approach, rationale | Page 7, line 140 – Page 7, line 156 |
| S15 | Techniques to enhance trustworthiness | Techniques to enhance trustworthiness and credibility of data analysis (e.g., member checking, audit trail, triangulation); rationale | Page 7, line 148-155 |
| **Results/findings** | | | |
| S16 | Synthesis and interpretation | Main findings (e.g., interpretations, inferences, and themes); might include development of a theory or model, or integration with prior research or theory | Results section, Page 7, line 163 – Page 14, line 335 |
| S17 | Links to empirical data | Evidence (e.g., quotes, field notes, text excerpts, photographs) to substantiate analytic findings | Quotes provided for each theme/sub-theme in results section |
| **Discussion** | | | |
| S18 | Integration with prior work, implications, transferability, and contribution(s) to the field | Short summary of main findings; explanation of how finding and conclusions connect to, support, elaborate on, or challenge conclusions of earlier scholarship; discussion of scope of application/generalizability; identification of unique contribution(s) to scholarship in a discipline or field | Discussion section, Page 15, line 336 – Page 19, line 434 |
| S19 | Limitations | Trustworthiness and limitations of findings | Strengths and Limitations Section, Page 19, line 435 – Page 20, line 462 |
| **Other** | | | |
| S20 | Conflicts of interest | Potential sources of influence or perceived influence on study conduct and conclusions; how these were managed | Page 21, line 480-485 |
| S21 | Funding | Sources of funding and other support; role funders in data collection, interpretation, and reporting | Title page – financial support and Page 20, line 478 - 479 |
