# Supplementary material for: Patients’ experiences of and roles in interprofessional collaborative practice in primary care: a constructivist grounded theory study
Source: Prim Health Care Res Dev. 2024 May 9;25:e24. doi: 10.1017/S1463423624000148 (PMC11091539; doi:10.1017/S1463423624000148)
Supplement: Davidson et al. supplementary material 2 — Davidson et al. supplementary material [file S1463423624000148sup002.docx]

Article Title: Patients’ experiences of interprofessional collaborative practice in primary care: a constructivist grounded theory study

Intro:

Hi, my name is [blinded for peer-review], I am a student from [blinded for peer-review]currently doing my PhD. Thank you for taking time out of your day to speak with me today. I am doing this research because I would really like patients to feel more able to be involved in in their own care and be provided with good healthcare.

In this research we are interested in Interprofessional Collaborative Practice, that sounds like a lot of words, but it is a term that is also sometimes used interchangeably with team care, multidisciplinary care, interdisciplinary care. But at the heart of it, it means multiple professionals working together with the patient and their family/support people to provide good health care in the community.

| Semi-Structured Interview Guide | | | |
| --- | --- | --- | --- |
| **No.** | **Type** | **Key question and prompts** | **Justification from literature, or research team discussion.** |
| 1 | Introductory questions – Ice breaker | 🡪 Can you start by telling me a bit about yourself?  Prompts:   - gender, age, geography/location, condition(s) - years lived with condition – for theoretical sampling, stage and complexity of condition, cultural background, Aboriginal or Torres Strait Islander identification | Davis, et. al. 2018 |
| 2 | Complex/Chronic Disease perspectives | 🡪 Could you tell me a bit about your medical history?  Could you tell me a bit about how your current health is managed?  Prompts:   - When were you first diagnosed with [condition]? - Who was involved in your care initially? Has this changed over the years/months? (For example: GP, practice nurse, allied health, specialists, etc.) - Who is involved in your care now? What professions? - Ask about family, friends and non-health professionals. - Do you perceive they are vital in your care? Why, why not? - What role do you perceive they play? - How were they introduced as part of your care? (For example: referral letters, care plans, My Health Record) | Davis, et. al. 2018  Banfield, et. al. 2017  Cheong, et. al. 2013 |
| 3 | Case Questions | 🡪 Could you tell me about a specific time you were more involved in your own treatment/care?  Prompts:   - Who else was involved? - To what level did you feel you were involved? - What was your role in this case? - What was the outcome of this case? - Have you been involved in a situation with multiple health professionals? (For example, shared medical appointments – with two or more professionals present) - What was the goal of this situation? Was this achieved?   🡪Could you tell me about a specific time you felt you were NOT involved or were excluded from your own treatment/care?  Prompts:   - Who was involved? - Do you know why you might not have been involved? - How did you feel not being involved? - Would you like to have been involved? - What was the outcome of this case? - What was the goal of this situation? Was this achieved? | van Dongen, et. al. 2017  Glasgow, et. al. 2005  Banfield, et. al. 2017 |
| 4 | Roles | 🡪What do you perceive to be your role in team care?   - How did you determine that was your role? - Are you satisfied with this role? - Could this role be different? Improved? - Could health professionals be more encouraging/supportive of this role? - How could you tell people about this role? | Cheong, et. al. 2013a Cheong et. al. 2013b  Cheong, et. al. 2015  Pullon, et. al. 2011 |
| 5 | IPCP | In your experience how would you define this type of care.  🡪How would you define IPCP?  When I say interprofessional collaborative practice (or team care, or multidisciplinary care) what sort of things come to mind for you? | van Dongen, et. al. 2017  Banfield, et. al. 2017 |
|  | Checking In | How do you feel after the interview? |  |
|  | Sampling/Recruitment | If not already identified/for clarification:  🡪Who is involved in your care?   - Provide their names, location of work and contact details?   We would like to speak to as many people about their experiences with their healthcare, do you have any family or friends who may be interested in being interviewed? | Allen, M. 2017 |

References for interview guide:

Allen, M. 2017. The SAGE encyclopedia of communication research methods, Sage Publications.

Banfield, M., Jowsey, T., Parkinson, A., Douglas, K. A. & Dawda, P. 2017. Experiencing integration: a qualitative pilot study of consumer and provider experiences of integrated primary health care in Australia. BMC Family Practice, 18, 2.

a Cheong, L. H., Armour, C. L. & Bosnic-Anticevich, S. Z. 2013. Multidisciplinary collaboration in primary care: through the eyes of patients. Australian Journal of Primary Health, 19, 190-7.

b Cheong, L. H., Armour, C. L. & Bosnic-Anticevich, S. Z. 2013. Primary health care teams and the patient perspective: a social network analysis. Research In Social & Administrative Pharmacy, 9, 741-57.

Cheong, L. H., Armour, C. L. & Bosnic-Anticevich, S. Z. 2015. Patient asthma networks: understanding who is important and why. Health expectations : an international journal of public participation in health care and health policy, 18, 2595-2605.

Davis, M. M., Gunn, R., Gowen, L. K., Miller, B. F., Green, L. A. & Cohen, D. J. 2018. A qualitative study of patient experiences of care in integrated behavioral health and primary care settings: more similar than different. Translational Behavioral Medicine, 8, 649-659.

Glasgow, R. E., Wagner, E. H., Schaefer, J., Mahoney, L. D., Reid, R. J. & Greene, S. M. 2005. Development and validation of the Patient Assessment of Chronic Illness Care (PACIC). Med Care, 43, 436-44.

Pullon, S., Mckinlay, E., Stubbe, M., Todd, L. & Badenhorst, C. 2011. Patients' and health professionals' perceptions of teamwork in primary care. Journal of primary health care, 3, 128-135.

van Dongen, J. J. J., Lenzen, S. A., Van Bokhoven, M. A., Daniëls, R., Van Der Weijden, T. & Beurskens, A. 2017. Interprofessional collaboration regarding patients' care plans in primary care: a focus group study into influential factors. BMC family practice, 17, 58.
