# Supplementary material for: Patients’ experiences of and roles in interprofessional collaborative practice in primary care: a constructivist grounded theory study
Source: Prim Health Care Res Dev. 2024 May 9;25:e24. doi: 10.1017/S1463423624000148 (PMC11091539; doi:10.1017/S1463423624000148)
Supplement: Davidson et al. supplementary material 3 — Davidson et al. supplementary material [file S1463423624000148sup003.docx]

Article Title: Patients’ experiences of interprofessional collaborative practice in primary care: a constructivist grounded theory study


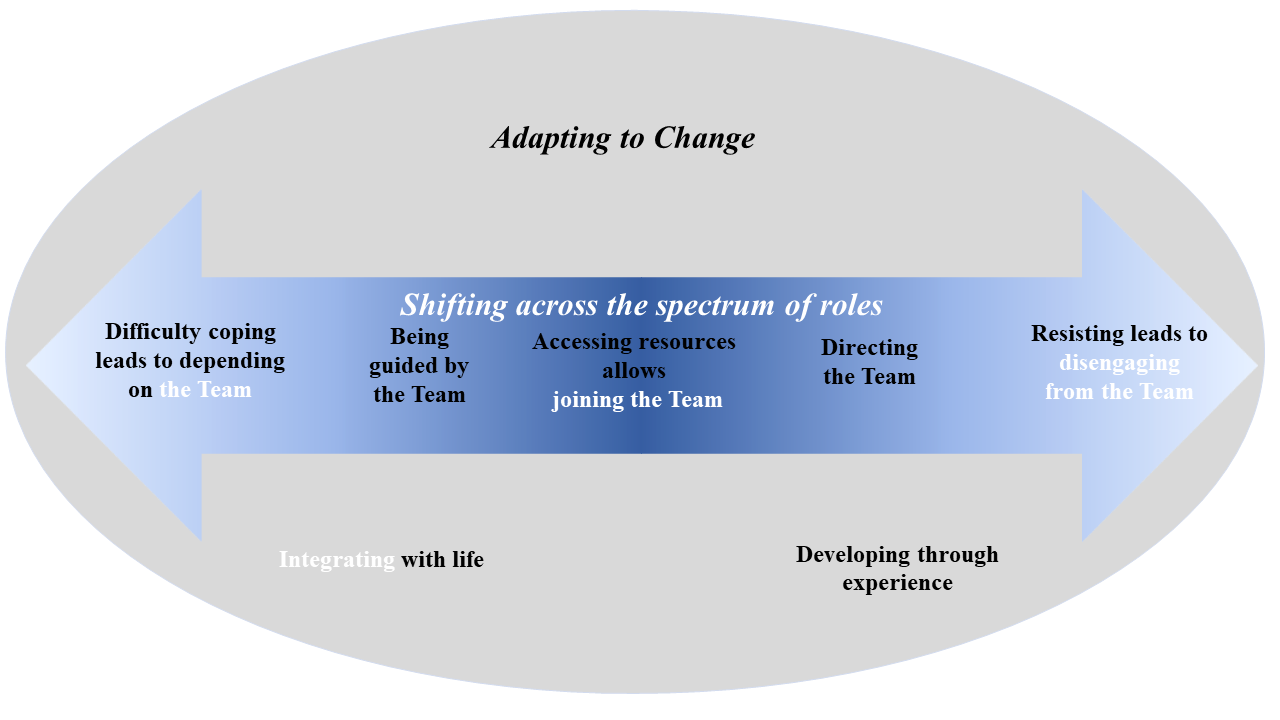

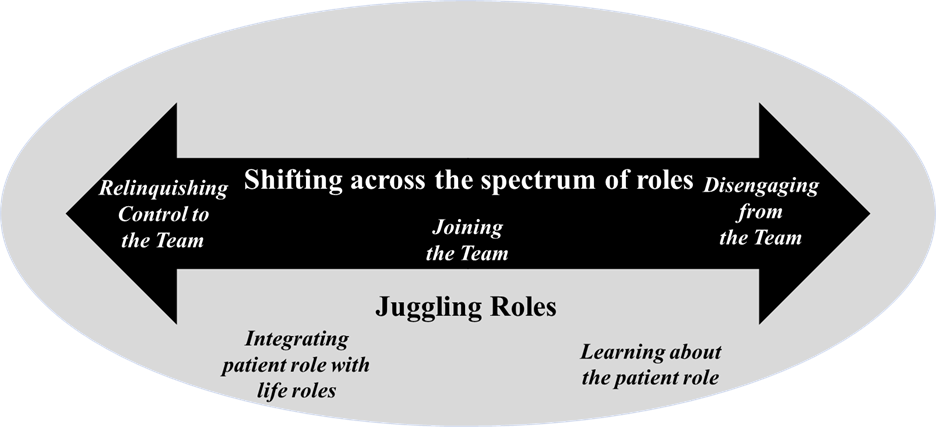


Patient advocate focus group study theoretical framework [1] Patients with chronic conditions interview study theoretical framework [2]

Figure 2: Comparison of patient advocate focus group study theoretical framework and adapted theoretical framework with results from patients with lived experience of living with a chronic condition.

[1] Davidson AR, Morgan M, Ball L, Reidlinger DP. Patient advocates' views of patient roles in interprofessional collaborative practice in primary care: A constructivist grounded theory study. Health Soc Care Community. 2022 Nov;30(6):e5775-e5785. doi: 10.1111/hsc.14009.

[2] Available on Figshare: [10.6084/m9.figshare.21588816](https://doi.org/10.6084/m9.figshare.21588816)
